# Geographical clustering of prostate cancer grade and stage at diagnosis, before and after adjustment for risk factors

**DOI:** 10.1186/1476-072X-4-1

**Published:** 2005-01-13

**Authors:** Ann Carroll Klassen, Martin Kulldorff, Frank Curriero

**Affiliations:** 1 Faculty of Social and Behavioral Sciences, Department of Health Policy and Management, Johns Hopkins Bloomberg School of Public Health, 624 N Broadway, Room 745, Baltimore, Maryland 21205, USA; 2 Department of Ambulatory Care and Prevention, Harvard Medical School and Harvard Pilgrim Health Care, 113 Brookline Avenue, 6^th ^Floor, Boston, Massachusetts, 02215, USA; 3 Department of Biostatistics, Johns Hopkins Bloomberg School of Public Health, Baltimore, Maryland 21205, USA

## Abstract

**Background:**

Spatial variation in patterns of disease outcomes is often explored with techniques such as cluster detection analysis. In other types of investigations, geographically varying individual or community level characteristics are often used as independent predictors in statistical models which also attempt to explain variation in disease outcomes. However, there is a lack of research which combines geographically referenced exploratory analysis with multilevel models. We used a spatial scan statistic approach, in combination with predicted block group-level disease patterns from multilevel models, to examine geographic variation in prostate cancer grade and stage at diagnosis.

**Results:**

We examined data from 20928 Maryland men with incident prostate cancer reported to the Maryland Cancer Registry during 1992–1997. Initial cluster detection analyses, prior to adjustment, indicated that there were four statistically significant clusters of high and low rates of each outcome (later stage at diagnosis and higher histologic grade of tumor) for prostate cancer cases in Maryland during 1992–1997. After adjustment for individual case attributes, including age, race, year of diagnosis, patterns of clusters changed for both outcomes. Additional adjustment for Census block group and county-level socioeconomic measures changed the cluster patterns further.

**Conclusions:**

These findings provide evidence that, in locations where adjustment changed patterns of clusters, the adjustment factors may be contributing causes of the original clusters. In addition, clusters identified after adjusting for individual and area-level predictors indicate area of unexplained variation, and merit further small-area investigations.

## Background

Ideally, contextual analysis allows for consideration of both attributes that are generalizable across multiple settings, and geographically referenced relationships – influences that occur in context with each other. However, a tension exists between geographic variation analysis, which identifies the location and nature of the variation, and non-spatial analysis, which may identify characteristics of environments or individuals associated with variation, but does so without spatially specific models.

Spatial variation in disease characteristics occurs, and multiple statistical methods have been developed to determine whether patterns of variation occur by chance alone, or whether variation is unlikely to have happened at random[1]. One type of variation analysis is cluster detection analysis, which specifically examines geographic clustering – spatial groups of outcomes that are statistically unlikely to occur by chance alone, given the overall distribution of the outcome of interest across the entire space being examined. Examples might be the occurrence of the disease itself [2,3], or distributions of factors of interest, such as characteristics of the disease, intermediate events such as extent of the disease at time of diagnosis [4] and receipt of certain treatments [5], or outcomes such as mortality related to the disease [6]. However, if clustering of an outcome is identified and determined to occur non-randomly, there is still little information on which to act, because the reasons for these clusters remain hidden.

Conversely, conventional non-spatial analysis methods may be used to identify important influences on individual or area-level disease variation. For example, hierarchical or multilevel regression can be used to simultaneously examine individual and area-level characteristics which are associated with variation in disease incidence, characteristics, or outcome [7]. However, these methods usually consider areas as discrete, even when they are contiguous, without examining relationships between the largest units of analysis. If areas in analyses are geographically related, after building multilevel models, it is still necessary to examine the data for spatial dependence, and to determine whether the model fully accounts for geographic patterns, or whether there is remaining unexplained variation that is spatially dependent – including, but not limited to, geographic clustering.

The study of disease patterns in prostate cancer, for example, can be informed by geographic analyses. Prostate cancer is a disease with strong geographic variation, both internationally and also within individual countries or regions [8]. Like most cancers, the development of prostate cancer typically occurs over a long period of time. Both age of onset and disease course vary enormously, but it has been demonstrated through autopsy study that most men will develop some degree of prostate cell abnormality in older age. It is likely that many factors contribute to its development; from inherited genetic risk, to lifestyle patterns in diet, use of substances such as tobacco and alcohol, exercise and body size and composition, to environmental exposures to a range of protective and detrimental agents [9]. Furthermore, although much is still unknown about prostate cancer etiology and development, there is sufficient information to argue that prostate cancer is most likely caused by a complex combination of factors, rather than a single explanatory risk. Beyond simple incidence, outcomes such as stage at diagnosis, tumor biology and histologic grade, receipt of standard-of-care treatment, and high quality survivorship are also geographically patterned.

When considering the utility of a geographic approach to prostate cancer influences, it may be useful to think of three broad categories of factors. There are factors which may be, at first consideration, purely non-geographic in influence. An example of this might be the influence of the biological characteristics of the cancer on the disease course, such as the relationship between histologic grade of tumor on the stage or extent of disease at diagnosis [10]. This relationship is considered important and tumor characteristics such as grade are almost always included when modeling outcomes. Yet we can consider this influence to be relatively non-geographic, because we might speculate that this relationship does not change under local geographic influences.

Other factors, such as age, might be considered to be pseudo-geographic in influence. The age distribution of the male population would vary across almost any geographic area under consideration, and there is also a strong age-disease relationship in prostate cancer, with the risk of the disease increasing with age. However, the age-disease relationship is not likely to be primarily driven by geography. Adjusting for the distribution of age within a population of interest is often desirable, in order to remove the confounding caused by age, and simulate the geographic variation we would expect to see if we had populations with identical age distributions.

A third and more complex category of influences are those for which geographic context is critical to their causal pathway, and thus these variables may be only partially understood outside of their geography. Examples might be individual social or behavioral characteristics such as ethnicity or race, income, insurance or education, occupation, diet or body size.

For example, the consistently greater risk for prostate cancer among men of African ancestry compared to all other ethnic groups in the world suggests fundamental biologic causes that supersede geographic influences. However, substantial geographic variation within the US African-American population, as well as international variation between African, Afro-Caribbean, and US men of African ancestry suggests complex multigenerational social and geographic influences [11].

Even influences that we may confidently classify as so fundamental as to be geographically immutable, such as the relationship between tumor biology and disease progression, could be influenced by geographic variation in access to care or medical practices, dietary, occupational, or environmental agents, or individual variation in behaviors such as tobacco use, exercise, or body size. Therefore, the extent to which any factor's influence on a cancer outcome varies by context or location offers tremendous insight into the mechanisms of influence.

The purpose of this research was to combine cluster detection analysis techniques with multilevel modeling of area-level influences on disease patterns, in order to examine the relationship between social-environmental influences and spatial patterning. We used data from the Maryland Cancer Registry on incident cases of prostate cancer occurring in Maryland from 1992 to 1997, and examined variation in two disease characteristics which contribute significantly to overall disease burden: histologic grade of tumor, and stage of disease at time of diagnosis. The use of geographic analysis of prostate cancer outcomes of interest, in combination with modeling of known risk factors, may prove useful in understanding how much of the strong geographic patterns in prostate cancer can be explained by individual and area-level influences, and how much remains, as of yet, unexplained.

For each of our two outcomes of interest, higher tumor grade and later stage of disease at diagnosis, we first modeled the "crude" or unadjusted variation in these outcomes across the entire State. This was done by calculating a block group-specific expected rate of each outcome, based simply on the number of cases within the block group and the overall rate of the outcome across the State, and comparing the ratio of observed to expected cases with the given outcome at the block group level. We then used estimates from multivariate models to refine our estimates of the expected number of higher grade or later stage cases, and recalculated, at the blockgroup level, the ratio of observed to expected cases with the outcome of interest. Throughout each set of three analyses, the observed number of cases remained the same, and the expected number (the denominator) varied with each adjustment. Therefore, if an independent variable in a regression model was positively associated with excess risk for the outcome of interest, it increased the regression-estimated expected number of such cases, and thus decreased the observed-to-expected ratio in areas where it was observed. Factors which were negatively associated with risk for the outcome, when adjusted for, reduced the number of such cases expected, and, in turn, increased the observed-to-expected ratio. The methods used are explained in greater detail in the methods section.

## Results

Table 1 describes the overall population of prostate cancer incident cases reported to the Maryland Cancer Registry during 1992–1997, as well as the population used for each analysis. Cases ranged in age from 16 to 106, with a median age of 69. Among cases retained for analysis, 26% were African-American. Overall, in Maryland during the time period 1992–1997, 23% of cases whose record contained histologic grade information had a tumor grade of 3 (poorly differentiated) or 4 (non-differentiated), and 21% staged cases had their disease detected after it had spread outside the prostate gland (stage 2 through 7).

**Table 1:** Characteristics of prostate cancer cases in Maryland, 1992–1997

	Registry Population N = 23993		Stage Analysis N = 19223		Grade Analysis N = 18947	
Age Group	n	%	n	%	n	%
16–49	403	2	352	2	325	2
50–69	11777	49	10228	53	9868	52
70–79	8739	36	6833	36	6853	36
80–106	3002	13	1810	9	1901	10
Missing	72	1	0	0	0	0
						
Race/Ethnicity						
White	16565	69	14255	74	14114	74
Black	5779	24	4968	26	4833	26
Asian	11	1	0	0	0	0
Native American	12	1	0	0	0	0
Other, Not Specified	343	1	0	0	0	0
Missing	1283	5	0	0	0	0
						
SEER Summary Stage at Diagnosis						
0	80	1	0	0	0	0
1	15679	65	15233	79	13798	73
2	2250	9	2190	11	2000	10
3	263	1	255	1	220	1
4	170	1	165	1	152	1
5	150	1	145	1	127	1
7	1274	5	1235	7	945	5
Missing	4127	17	0	0	1705	9
						
Grade at Diagnosis						
1	2505	10	2042	10	2289	12
2	13112	55	11301	59	12335	65
3	4425	18	3786	20	4199	22
4	128	1	113	1	124	1
Missing	3823	16	1981	10	0	0

**Figure 1:**
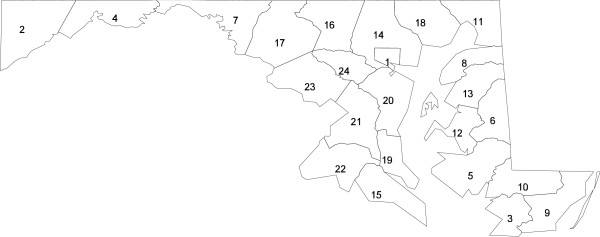
**Maryland counties, ranked by county resource index score**. Maryland's 24 counties ranked from lowest (Baltimore City) to highest (Howard County), based on combined score on four 1990 US Census population characteristics (table 2).

Figure 1 and table 2 provide information on the four-item county-level social resource index used in the multilevel analysis. The six suburban counties surrounding Washington, D.C. have the highest scores on this index, with Baltimore City and the rural areas of western Maryland and the Eastern Shore of the Chesapeake Bay region having the lowest scores. Both low and high scoring counties contribute substantial numbers of African-American cases to the analysis.

**Table 2:** County resource index score and subcomponents, Maryland 1990 Census

County	County Resource Index Score^1^	Index Subcomponents: 1990 Census				# Cases	% Cases Who Are Black
				
		% High School Graduate^2^	% Employed^3^	% Moved in last 5 years^4^	Median Household Income ($1000)^5^		
1. Balto. City	-1.58	61	91	42	24	3645	61
2. Garrett	-1.57	68	93	34	23	109	1
3. Somerset	-1.54	61	92	42	23	100	33
4. Allegany	-1.48	71	92	36	22	480	1
5. Dorchester	-1.41	65	94	38	25	202	33
6. Caroline	-1.01	67	96	42	28	161	24
7. Washington	-.72	69	96	45	30	517	3
8. Kent	-.68	71	97	43	30	104	32
9. Worcester	-.57	71	95	50	28	247	21
10. Wicomico	-.47	72	95	51	29	300	25
11. Cecil	-.37	72	95	45	36	256	7
12. Talbot	-.34	77	98	44	32	286	15
13. Queen Anne's	-.07	77	96	45	39	199	18
14. Balto. Co.	-.06	78	96	43	39	3890	11
15. St. Mary's	.11	77	96	51	37	202	21
16. Carroll	.11	79	97	43	42	605	4
17. Frederick	.36	80	97	49	41	497	7
18. Harford	.38	82	97	49	42	760	9
19. Calvert	.45	79	97	47	48	197	20
20. Anne Arundel	.52	81	97	49	45	1671	13
21. Prince Geo	.55	83	96	51	43	2457	52
22. Charles	.55	81	97	49	46	350	33
23. Montgomery	1.41	91	97	53	54	3077	11
24. Howard	1.59	91	98	57	54	618	19

1. County resource index scores were calculated by summing the raw score of four measures (percent high school graduates, percent employed, percent moved in last 5 years, and median household income), subtracting the mean of the raw composite scores, and dividing by the standard deviation of the raw composite scores. 2. Percent of persons 25 years or older who have received a high school diploma or its equivalent (e.g. GED) or higher (e.g. college or professional school). 3. Percent of persons 16 years old and over in the labor force who are currently employed. 4. Percent of residents age 5 and older who were not living in the same dwelling five years ago.5. Summed incomes (in thousands of dollars) of household members 15 years of age and older.

### Cluster detection results – higher grade of tumor

**Figure 2:**
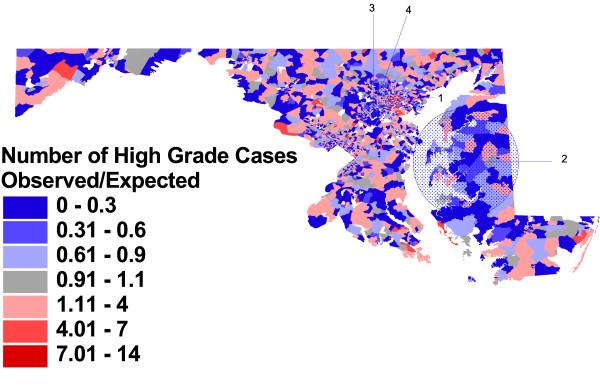
**Observed vs. expected block group rates of high grade tumors, and significant clusters**. Proportion of prostate cancer cases with histologic grade of 3 or 4, compared to proportion expected based on overall Maryland rate, Maryland Cancer Registry, 1992–1997, N = 18949. A spatial scan statistic was used to identify non-overlapping clusters of statistically significant high or low rates (table 3).

**Figure 3:**
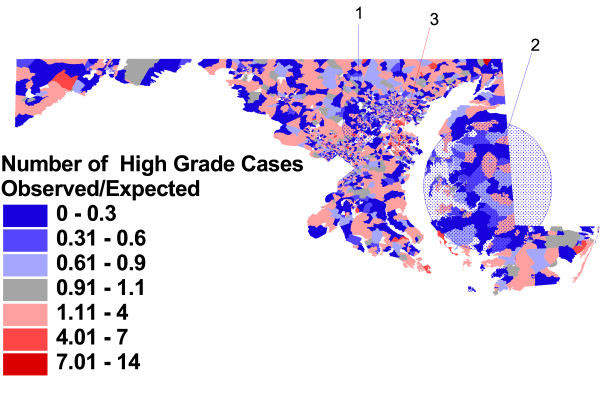
**Observed vs. expected block group rates of high grade tumors, adjusted for case characteristics, and significant clusters**. Proportion of prostate cancer cases with histologic grade of 3 or 4, compared to proportion expected based on case characteristics of age, race and year of diagnosis.

**Figure 4:**
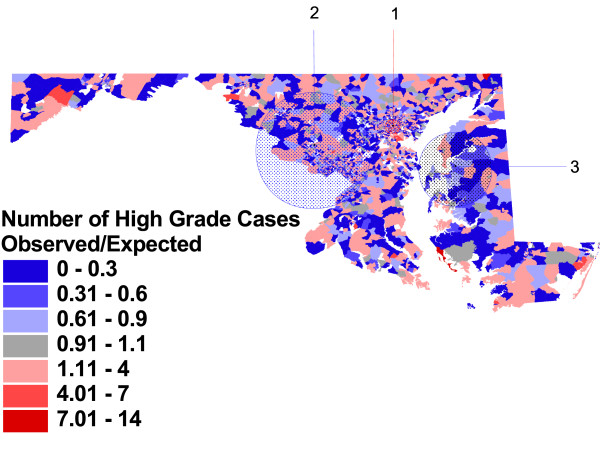
**Observed vs. expected block group rates of high grade tumors, adjusted for case and area-level characteristics, and significant clusters**. Proportion of prostate cancer cases with histologic grade of 3 or 4, compared to proportion expected based on case characteristics of age, race and year of diagnosis, and area-level Census characteristics.

Figures 2, 3, and 4, and the related table 3, show the block group-level patterns of tumor histology across the State. Of the 3670 Maryland 1990 Census block groups, 3313 (90%) contained cases used in this analysis; the number of cases per block group ranged from 1 to 99 with a median of 4.

**Table 3:** Cluster Analysis of Higher Grade* Prostate Cancer Cases – Maryland Cancer Registry, 1992–1997

	Radius (km)	# Block groups in Cluster	# Higher Grade Cases Expected	# Higher Grade Cases Observed	Relative Risk	P Value
Map 2. Unadjusted Analysis						
Cluster 1	5.99	550	522.5	669	1.28	.001
Cluster 2	44.93	201	305.3	210	0.69	.001
Cluster 3	10.34	173	253.9	176	0.69	.004
Cluster 4	5.93	38	93.8	49	0.52	.017
Map 3. Adjusted Analysis **						
Cluster 1	14.81	292	487.3	362	0.74	.001
Cluster 2	54.65	162	247.6	164	0.66	.003
Cluster 3	8.27	80	99.6	156	1.56	.013
Map 4. Adjusted Analysis ***						
Cluster 1	6.02	554	444.0	643	1.45	.001
Cluster 2	48.62	1181	1825.4	1587	0.87	.001
Cluster 3	30.88	99	155.8	94	0.60	.004

* Among cases with a histologic grade, those cases graded as 3 or 4 vs. 1 or 2.

** Expected Rate Adjusted for Race, Age, Year of Diagnosis.

*** Expected Rate Adjusted for Age, Race, Year of Diagnosis, and Area-Level Census Characteristics.

Figure 2 shows that most block groups vary from the expected proportion of high grade cases (23%); block groups with lower proportions of high grade cases are displayed in blue, and those with greater than expected rates of high grade cases are shown in red. (Block groups contributing no cases to the analysis are identified in white on the maps.) Much of this variation is random, and not statistically different than we would expect by chance. Furthermore, because a block group's spatial size is inversely proportional to population density, large individual block group areas of deep color, although striking to the eye, are unlikely to include a substantial proportion of the case population, and therefore would not constitute a statistically significant area of variation on their own. However, figure 2 identifies four non-overlapping clusters with statistically significant (p < .05) higher or lower rates of aggressive grade.

The most likely cluster is a geographically small densely populated area in Baltimore City, with a relative risk (RR) of 1.28 (p = .001). The second most likely non-overlapping cluster is a large area in the center of the Eastern Shore of the Chesapeake Bay, with a significantly lower rate of high grade tumors in men with prostate cancer (RR = 0.69, p = .001). Two small areas of lower rates in the suburban areas outside of Baltimore City were identified, one to the north of the City (RR = 0.69, p = .004) and one to the southwest (RR = 0.52, p = .017).

Figure 3 shows that adjustment for individual case characteristics (older age, black race, and earlier year of diagnosis) changes the number and location of statistically significant clusters of high and low rates of aggressive grade. The most likely cluster is an area of lower risk for aggressive grade located between Baltimore City and Washington DC (RR = 0.74, p = .001); this area overlaps with the area contained in cluster 3 in figure 2. Similarly, a large area of the Eastern Shore is again identified as the second most likely cluster with a lower relative risk for higher grade tumors (RR = 0.66, p = .003). There are no longer significant non-overlapping clusters in Baltimore City or northwestern Baltimore County. However, a previously non-significant area of excess risk in Anne Arundel County is now identified, based on the number of cases expected from individual case risk characteristics, as having statistically significant excess risk (RR = 1.56, p = .013). This cluster was identified but not reported due to borderline statistical significance (p = .09) in the unadjusted analysis (figure 2).

Figure 4 shows results of a cluster detection analysis comparing the observed and expected numbers of cases of high grade tumor in each block group, based on individual case characteristics, and also block group and county-level population characteristics (block group median household income, as well as the composite index of county-level high school attainment, employment, income, and residential mobility). Adjusting for these area-level social influences changes both the number and location of block groups found to have higher or lower rates than expected by chance.

The most likely cluster in this analysis is an area of higher than expected rates of aggressive tumor among cases, located to the west of Baltimore City (RR = 1.45, p = .001). This small area was previously identified as the most likely cluster in figure 2, but with a lower relative risk, and was not identified as having higher rates than expected in the analysis adjusting for individual characteristics (figure 3). The second most likely cluster in this analysis is a large area of lower than expected rates (RR = 0.87, p = .001), located in several counties to the north and west of Washington DC. This area includes small clusters 3 from figure 2 and cluster 1 from figure 3, but the majority of block groups in this cluster were not previously included in the clusters found in the previous analyses. The third most likely cluster in this analysis is located on the Eastern Shore, and although it includes areas identified in the two previous clusters detected on the Eastern Shore, it is both smaller in area and has lower estimate of relative risk for aggressive disease among cases in this area (RR = 0.60, p = .004).

### Cluster detection results – stage at diagnosis

**Figure 5:**
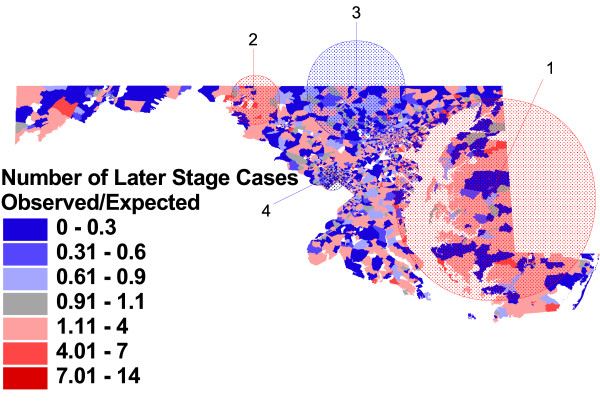
**Observed vs. expected block group rates of later stage at diagnosis, and significant clusters**. Proportion of prostate cancer cases with stage of disease at diagnosis of 2 to 7, compared to proportion expected based on overall Maryland rate, Maryland Cancer Registry, 1992–1997, N = 19223. A spatial scan statistic was used to identify non-overlapping clusters of statistically significant high or low rates (table 4).

**Figure 6:**
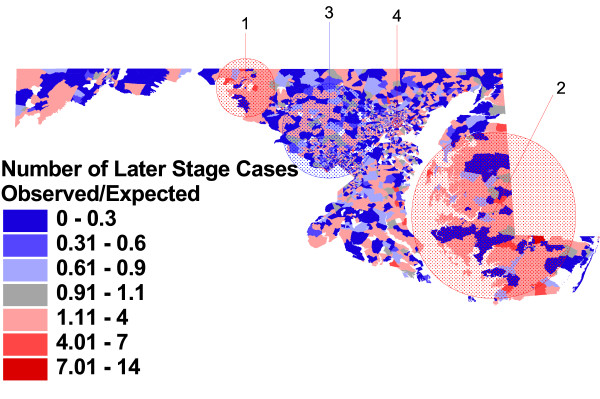
**Observed vs. expected block group rates of later stage at diagnosis, adjusted for case characteristics, and significant clusters**. Proportion of prostate cancer cases with stage of disease at diagnosis of 2 to 7, compared to proportion expected based on case characteristics of age, race, tumor grade, and year of diagnosis.

**Figure 7:**
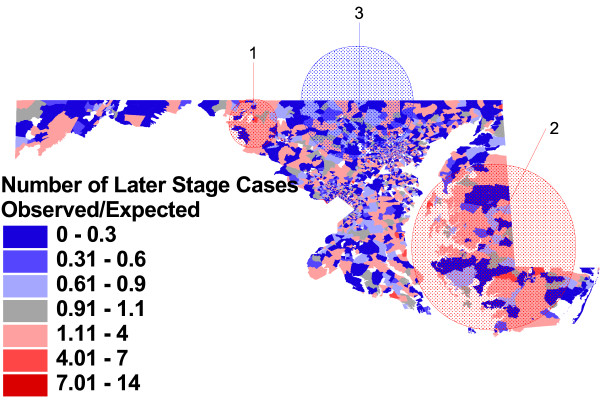
**Observed vs. expected block group rates of later stage at diagnosis, adjusted for case and area-level characteristics, and significant clusters**. Proportion of prostate cancer cases with stage of disease at diagnosis of 2 to 7, compared to proportion expected based case characteristics of age, race, tumor grade, and year of diagnosis, and area-level Census characteristics.

Figures 5, 6, and 7, and the related table 4, display results of the cluster detection analysis for later stage diagnosis. Cases were located in 90% (3313/3670) of Maryland's 1990 Census block groups; cases per block group ranged from 1 to 90 with a median of 4.

**Table 4:** Cluster Analysis of Later Stage* Prostate Cancer Cases – Maryland Cancer Registry, 1992–1997

	Radius (km)	# Block groups in Cluster	# Later Stage Cases Expected	# Later Stage Cases Observed	Relative Risk	P Value
Map 5. Unadjusted Analysis						
Cluster 1	85.32	1436	1481.0	1743	1.18	.001
Cluster 2	20.72	88	93.8	182	1.94	.001
Cluster 3	41.92	291	512.7	366	0.71	.001
Cluster 4	10.61	316	455.8	325	0.71	.001
Map 6. Adjusted Analysis **						
Cluster 1	24.71	96	98.3	191	1.94	.001
Cluster 2	69.96	326	372.5	533	1.43	.001
Cluster 3	39.82	1208	1633.4	1398	0.86	.001
Cluster 4	4.12	286	248.4	329	1.32	.029
Map 7. Adjusted Analysis ***						
Cluster 1	20.65	95	104.9	188	1.79	.001
Cluster 2	69.96	326	394.8	533	1.35	.001
Cluster 3	47.90	676	1014.0	831	0.82	.001

* Among cases receiving staging, those cases diagnosed at Stages 2–7 vs. Stage 1.

** Expected Rate Adjusted for Race, Age, Grade, and Year of Diagnosis.

*** Expected Rate Adjusted for Age, Race, Grade, Year of Diagnosis, and Area-Level Census Characteristics.

Statistically significant clusters of high or low rates were identified in four geographic areas in the unadjusted analysis (figure 5). As described in detail in table 4, the most likely cluster is the largest, covering most of the Eastern Shore and some of the adjacent Western Shore of the Chesapeake Bay region of Maryland, with cases in this area have a modestly elevated relative risk of later stage diagnosis (RR) = 1.12, p = .001). A smaller geographic area in the western area of the State was identified as the second most likely cluster, with a relative risk of 1.94 (p = .001). Two relatively affluent areas of the State were identified has having lower probability of later stage diagnosis: Montgomery County, a suburb of Washington D.C. (RR = 0.71, p = .001), and the suburban and rural areas to the north and west of Baltimore City (RR = 0.71, p = .001).

Figure 6 shows that, after adjusting for individual case attributes associated with late stage (black race, younger age, aggressive or missing tumor grade, and earlier year of diagnosis), the relationship between the observed number of later stage cases and the expected number changes in many block groups across the State. Although the visual pattern remains similar, the location and size of statistically significant clusters, as well as the relative risk of late stage diagnosis within those clusters, changes. The most likely cluster is now in western Maryland, with a relative risk which is essentially unchanged by adjustment for individual case characteristics (RR = 1.94, p = .001). The largest cluster has now been reduced in size and includes primarily the lower Eastern Shore, but the estimate of relative risk for later stage diagnosis in this area has increased (RR = 1.43, p = .001). The area of lower risk for cases in suburban Washington DC has grown to include much of the suburban area between Washington and Baltimore (RR = 0.86, p = .001), and a new area, centered in Baltimore City, has been identified as having greater risk for later stage diagnosis (RR = 1.32, p = .029).

Figure 7 displays results of a cluster detection analysis for later stage diagnosis, comparing actual counts to those expected when considering both individual men's age, race, year of diagnosis, and tumor biology, as well as their immediate neighborhood and county level of social resources – including occupation, education, employment, poverty and residential mobility. These additional adjustments change both the visual patterning of higher and lower rates, as well the location and estimates of relative risk for the statistically significant clusters identified. Two clusters of higher than expected rates of later stage diagnosis remain, covering essentially the same areas as in figure 6. The relative risk for later stage diagnosis in western Maryland has been reduced only slightly, from 1.94 to 1.79 (p = .001), and the relative risk for the secondary cluster on the Eastern Shore has been reduced from 1.43 to 1.35 (p = .001). Both the Baltimore City cluster and the suburban Washington DC clusters seen in the first two maps are no longer identified as statistically significant. However, a large area in the north central part of the State has been identified as having lower than expected rates of later stage diagnosis, with a relative risk of 0.82 (p = .001).

## Discussion

These geographic analyses provide information on both biological influences on cancer, as well as those more closely influenced by patterns of medical care. For tumor biology, the results of the unadjusted analysis suggest that one primarily rural area of the State, as well as two affluent suburban areas, appear to offer protection from high grade tumor histology. On the other hand, the urban Baltimore area has higher than expected rates of high grade tumors among men diagnosed in the time period 1992–1997.

Individual case characteristics change this picture dramatically, but do not "explain away" all variation in this important disease characteristic. For example, black race is an important risk factor for aggressive tumor biology; therefore, it is reasonable to speculate that area differences in the proportion of African-American men in the case population may have accounted for some of the clustering in figure 2, with clusters in primarily white northern Baltimore County and primarily black Baltimore City no longer statistically significant with race adjustment. Figure 3 shows that, despite individual case differences accounted for with adjustment, there are still three areas of the State with unusually high or low rates of aggressive disease.

Figure 4 shows some impact of further adjustment for social resource composition within small areas (block groups) and larger areas (counties). The interpretation of this adjustment is more speculative than confirmatory, but suggests some avenues for further research.

Large areas of the rural Eastern Shore of Maryland are no longer identified as being contained inside non-overlapping areas of statistically significant lower risk for aggressive tumor biology, with the protected area being narrowed from a radius of 54.65 kilometers to 30.88 kilometers. Conversely, the small protective area in affluent Howard County between Washington and Baltimore has now grown from a radius of 14.81 kilometers in figure 3 to 48.62 kilometers in figure 4. Anne Arundel County is no longer at excess risk but Baltimore City is.

The influence of area level social resources on high grade of tumor was complex: the men with the lowest risk for aggressive tumors were white men living in small areas of greater income, nested within counties of overall low social resources. Therefore, clusters remaining in figure 4 are those whose rates are either higher or lower than expected given their social characteristics.

The cluster in Baltimore City reflects the fact that Baltimore City does not fit the overall model of low resource counties as protective. Baltimore City is the single urban county in the lowest range of the index; the rest of the lower resource counties are predominantly rural. Therefore, moving from figure 3 (only individual adjustment) to figure 4 (area-level adjustment) identified that Baltimore City's low social resources are not protective, to same effect as in rural counties. This difference may be caused by any number of lifestyle differences between urban and rural low income communities. Although individual case race is not likely to be driving this difference, it may be that area-level racial composition is another piece of this puzzle, given that Baltimore City's racial composition differs so dramatically from the other low resource counties.

Conversely, the protective clusters are found in counties with high social resource index scores, centered in Montgomery County in the Washington, D. C. suburbs, and in an area with slightly low scores, Talbot and Queen Anne's counties on the Eastern Shore. For the D.C. suburbs, their rate in figure 2 is neither high nor low; however, their high social resource index score would predict high rates; therefore they create a lower- than-expected cluster.

For the Eastern Shore, the lower rate of aggressive disease has been consistent across all three cluster analyses. For low social resource counties such as Dorchester, the adjusted predicted rate in figure 4 is now consistent with expected low rates, and therefore this area is no longer part of a cluster. However, the rate is lower than expected in counties with slightly higher resources, and therefore the most affluent Eastern Shore counties (Talbot and Queen Anne's) continue to be identified as lower than expected.

Finally, Anne Arundel County, which had higher than expected rates in the individually adjusted analysis, is now no different than expected, arguing that the relatively high social resource index score for this county led to a closer approximation of expected proportion of cases with aggressive disease.

When considering the geographic patterning of later stage at diagnosis for men with prostate cancer in Maryland during the time period 1992 to 1997, it appears from the unadjusted analysis that men in certain rural areas were much more likely to come into treatment with more advanced disease than those in the suburban, more affluent areas of the State. Individual characteristics of the patients appear in some ways to have masked these geographic differences, in that the clusters generally remain or become more important once the case population mix of characteristics such as age, race, tumor biology, and year of diagnosis is taken into account (figure 5 versus figure 6). Additionally, an area of Baltimore City, which has a greater proportion of young, African American men than the rest of the State, became significantly more likely to have later stage cases, after adjustment for age and race. This suggests that men in Baltimore are specifically disadvantaged in terms of early detection of disease, beyond what would be predicted by their age, race, or tumor biology.

From figure 7, we see evidence that area-level socioeconomic resources may contribute to these patterns. The relative risk for late stage diagnosis in the two rural clusters has been reduced somewhat, and the cluster of lower risk in the north-central part of the State is less different than in the unadjusted map. Baltimore City and the Washington suburbs no longer differ significantly from the rest of the State, supporting a socioeconomic influence on the previous clusters.

Prostate-specific antigen (PSA) testing was widely available in Maryland during the entire time period of this study (1992–1997). This suggests that more global barriers to health care, rather than differential access to this specific diagnostic tool, were more important in creating these patterns of late stage diagnosis.

## Conclusions

Although there is no statistical test to evaluate the proportion of variation explained in a multilevel model, because of the inclusion of random effects, it is reasonable to state that, overall, our adjustment methods did account for substantial variation in rates of aggressive disease and late stage diagnosis, by considering important influences – characteristics of the men themselves, as well as characteristics of their environments.

In spite of this, variation in these cancer characteristics remained substantial across the State. In fact, whether the measure used is the number of cases, number of block groups, or geographic area, figures 4 and 7 identify as much, if not more, deviation from the predicted pattern than the unadjusted figures 2 and 5 respectively. Many areas are included in clusters across all three analyses of a specific outcome. Given that the exact boundaries of clusters are always approximate, and would be expected to vary from analysis to analysis, it is important to note similarities as well as differences within each outcome-specific set of maps. This suggests that there are underlying causal influences that remain, despite the important relationships with the measures used for adjustment.

An additional caveat in the interpretation of these cluster detection analyses is related to the choice of criteria used for reporting clusters. In consideration of the large amount of data being examined, both in terms of geographic area and number of cases, we chose to report clusters only if they had a statistical significance of p < .05, and contained no geographic overlap with a more significant cluster. These restrictions meant that a given geographic area could possibly be described as part of a cluster of excess or reduced risk in one analysis, and not in other, based on small changes in expected number of cases, or based on the identification of a more significant cluster nearby.

These findings have implications on both a practical cancer control level, as well as for further research in prostate cancer. For state and local health agencies, trends in area-level patterns of cancer outcomes over time can be used to monitor change, whether to evaluate the effectiveness of geographically distributed interventions such as screening or treatment programs, or identify population changes which may increase need for services. Unadjusted cluster analyses provide valuable information for cancer control planners who need to address areas of greatest need, regardless of the cause. However, adjusted analyses identify geographically unique situations, such as the persistently elevated rates of later stage diagnosis in two rural areas of Maryland. For researchers, analytic techniques which identify both explained and unexplained geographic variation may provide information about the multilevel synergistic factors influencing cancer patterns, or, at a minimum, identify areas and populations meriting further study.

## Methods

### Data and data sources

More detailed information on these data and methods has been reported previously [10]. With IRB approval from the Johns Hopkins School of Public Health and the Maryland Department of Health and Mental Hygiene, and a data agreement between the two institutions, we obtained all incident cases of prostate cancer reported to the Maryland Cancer Registry during the years 1992–1997 (n = 24,189). Based on case residence address, we geocoded cases to latitude and longitude coordinates. For cases unable to be geocoded, we assigned cases to a coordinate location within their zipcode using a weighted imputation algorithm, based on 1990 US Census race-, age-, and gender-specific population distributions within their zipcode [12]. We thus assigned each nongeocoded case to a Census block centroid, based on the best-known distribution of men like himself within his zipcode.

Of 24,189 cases, 23,993 had verifiable Maryland addresses. Ninety-one percent (21,904) were successfully geocoded, and nine percent (2,089) were assigned to an imputed location within their zipcode by algorithm. An additional 3063 cases were not used, due to missing demographic or clinical data, or because their race was neither African-American or white, leaving a final analysis population of 20,928.

We used individual case characteristics from the Registry record, including age at diagnosis, race, year of diagnosis, tumor stage, and tumor histologic grade. Based on case residence, we added to each case record selected 1990 US Census characteristics of three nested geographic units surrounding the case location – the Census block group, Census tract, and county. Our record for each case therefore contained individual demographic and clinical characteristics based on the Cancer Registry data, point location of residence, and Census measures for the case's block group, tract and county of residence.

We created seventeen possible area-level social indicators for each block group, tract, and county from the 1990 US Census STF-3 file [13]: three measures of housing resources (median sale price, percent of owner occupied units, and housing value percentile rank, based on both rental and sale values, weighted by the proportions of rental and owner-occupied housing in an area), three income measures (median household income, median family income, and median per capita income), four population composition measures (percent white, percent black, percent born outside the US, and percent age 5 or older not living in the same residence for at least five years), two social class measures (percent high school graduates among persons age 25 and older, and percent employed in white collar jobs, defined as Census job classifications of managerial, professional, technical, sales, and administrative support) and five material deprivation measures (percent households without a car, percent households without a telephone, percent persons 16 and older who were unemployed, percent persons living in "crowded" residences, defined as more than one person per room, and percent of persons living in poverty).

For continuous variables (case age and census measures), we compute standardized measures to reduce collinearity, by centering each case value at the population mean, and dividing by the standard deviation. For year of diagnosis, we centered the values at 1994, a midvalue in our six year time window.

We chose two outcomes of interest which are associated with differences in prostate cancer disease severity and longterm survival for patients – histologic grade, or degree of cell differentiation, of the tumor, and stage, or extent, of disease at time of diagnosis. For each outcome, we dichotomized the data and examined the likelihood of the more negative outcome. For tumor grade, we compared tumors staged as 3 or 4 (poorly differentiated or undifferentiated) to those graded as 1 or 2 (well or moderately well differentiated). For stage at diagnosis, we used the Surveillance, Epidemiology and End Stage (SEER) summary stage [14], and compared cases diagnosed at stages 2 through 7 (regional to distant metastases) to those diagnosed at stage 1 (localized disease). Cases only missing staging information were retained for analyses of grade, and cases only missing grade were used in the analysis of stage at diagnosis. Because tumor histology is an important predictor of rapidly spreading disease, tumor grade was used as an independent predictor in modeling late stage disease.

### Cluster detection methods

In order to explore the geographic patterns of our two outcomes of interest, we used the spatial scan statistic [15] to detect and evaluate the statistical significance of any geographic clusters of each outcome. This method imposes a very large number of overlapping circles of different location and size on the map, each of which is a potential cluster, and adjusts for the multiple testing inherent in the many circles considered.

Our cluster detection method identified clusters of both high and low rates, with a maximum scanning window size to include up to 50% of the population at risk. Secondary clusters were reported if they had no geographic overlap with more likely clusters. P-values were derived from 999 simulated Monte Carlo replications under the null hypothesis of spatial randomness of outcomes of interest.

We conducted three separate cluster detection analyses for each of the two prostate cancer outcomes: higher histologic grade of tumor, and later stage at diagnosis. In the unadjusted analysis, under the null hypothesis, the expected number of more aggressive grade or late stage cases in a block group was calculated by multiplying the total case population of the block group by the statewide rate of the outcome of interest. Thus, in the unadjusted analysis, a block group would be expected to have the same rate or proportion of late stage or high grade cases in its case population as the State. In the two adjusted analyses, the expected number of aggressive grade or later stage cases was calculated from a regression model containing individual case characteristics, or from a regression model with both individual and area-level covariates. Based on the expected counts, the number of aggressive grade and later stage cases in each block group was modeled as a Poisson distribution.

For the unadjusted analyses, we also used a Bernoulli model to compare the distribution of so-called "cases" (those with aggressive grade or late stage) to "controls" (less aggressive grade or early stage) based on point location of each residential address, rather than rates within block groups. This was useful to compare the sensitivity of the Poisson model assumption for aggregated data to that of the unaggregated Bernoulli method. No major differences in results were found, and to allow proper comparison between the adjusted and unadjusted analyses, the Poisson model results are presented for all three types of analyses.

For each cluster identified, we list the radius, number of block groups in the cluster, the observed versus expected number of late stage or aggressive grade cases, the relative risk and the p value. The relative risk is the risk of the respective outcome within the cluster, compared to the population's risk. We report clusters with statistical significance p < .05 that do not overlap with another reported cluster with a lower p-value. Calculations were done using the freely available SaTScan v4.0 software .

### Sources of expected population counts

We used the results of two multivariate modeling methods to calculate the expected count of aggressive grade and late stage cases in each block group. In prior work [10] we built multivariate hierarchical logistic regression models [16,17] to identify individual and area-level factors significantly associated with aggressive grade and late stage among cases, and these findings, summarized below, served as the basis for calculating our expected population in each block group.

In logistic regression models including only individual level predictors, our final model included the following statistically significant associations with higher histologic grade of tumor: older age (Odds Ratio (O.R.) 1.17, 95% Confidence Interval (C.I.) 1.13, 1.21), black race (O.R. 1.46, 95% C.I. 1.35, 1.57), more recent year of diagnosis (O.R. 0.92, 95% C.I. 0.90, 0.94), and an interaction between age and year of diagnosis (O.R. 1.06, 95% C.I. 1.04, 1.08).

To build the multilevel models, we tested each of the 17 area-level indicators at each level, starting with block group, and also tested for interactions at each level and between levels. To avoid unstable models, when we found multiple significant Census predictors, we computed and tested simple indices by summing relevant Census measures. We also tested for random effects, to account for additional variability. In a multilevel logistic regression model of aggressive tumor grade, each of the above individual level variables remained significant. In addition, two area-level indicators were significant in the final model: block group median household income (O.R. 0.92, 95% C.I. 0.87, 0.96), with an interaction between black race and income (O.R. 1.12, 95% C.I. 1.02, 1.223), and a standardized county resource index, composed of four summed county-level measures: percent high school graduates, percent employed, percent moved within the past five years, and median household income (in $1000 units) (O.R. 1.23, 95% C.I. 1.16, 1.31). Random intercept terms were found to be significant at the block group and county level.

In logistic regression models including only individual level predictors, our final model included the following statistically significant associations with late stage at diagnosis: older age (O.R. 0.85, 95% C.I. 0.82, 0.90), black race (O.R. 2.97, 95% C.I. 1.35, 1.59), higher tumor grade (O.R. 2.97, 95% C.I. 1.35, 1.59), missing tumor grade (O.R. 5.56, 95% C.I. 2.77, 3.17), more recent year of diagnosis (O.R. 0.83, 95% C.I. 0.78, 0.88) and interactions between age and black race (O.R. 1.18, 95% C.I. 1.09, 1.27), grade and year of diagnosis (O.R. 1.06, 95% C.I. 1.02, 1.10), and missing grade and year of diagnosis (O.R. 1.19, 95% C.I. 1.10, 1.30).

In the multilevel logistic regression model of late stage at diagnosis, each of the above individual level variables remained significant. In addition, two area-level indicators were significant in the final model: block group percentage of white collar workers among the employed population (O.R. 0.93, 95% C.I. 0.89, 0.98), and the standardized county resource index (O.R. 0.94, 95% C.I. 0.89, 0.98). A statistically significant interaction existed between county resource score and older age (O.R. 0.95, C.I. 0.92, 0.99), and random intercept terms were found to be significant at the block group and county level.

### Calculation of block group-specific predicted populations

The models described above were used to calculate an expected count of aggressive grade and late stage cases, respectively, for each block group. This was accomplished by taking the inverse logit transform of the expected linear predictor in each logistic regression model, yielding a set of estimated probabilities for each outcome. These probabilities were then aggregated to the block group level, providing expected block group-specific counts of later stage and aggressive grade cases.

By definition the expected linear predictors includes only estimates from fixed effects in each multilevel logistic regression model. The random effects at the block group and county level, although influential on parameter estimation, were not included in these calculations. Compared to the unadjusted results, geographic patterns shown in the adjusted analyses could be interpreted as those existing after controlling for individual and area-level factors, respectively. In essence, this approach explores residual geographic variation. For this reason, information from the random effects is not included in determining expected outcome counts.

## Authors' contributions

AK obtained funding and data for this research, and was PI on the project. She conceptualized and conducted the analysis, and drafted the manuscript. MK directed the development and interpretation of the spatial analysis. FC worked with AK to develop and interpret the multilevel models used, as well as the methodology for imputation. All authors participated in the preparation and approval of the final version of the manuscript.
